# The Transcriptome of *Brassica napus* L. Roots under Waterlogging at the Seedling Stage

**DOI:** 10.3390/ijms14022637

**Published:** 2013-01-28

**Authors:** Xiling Zou, Xiaoyu Tan, Chengwei Hu, Liu Zeng, Guangyuan Lu, Guiping Fu, Yong Cheng, Xuekun Zhang

**Affiliations:** Key Laboratory of Biology and Genetic Improvement of Oil Crops, Ministry of Agriculture, Oil Crops Research Institute of the Chinese Academy of Agricultural Sciences, Wuhan 430062, China; E-Mails: zouxiling@gmail.com (X.Z.); tanxy85@163.com (X.T.); chw63@126.com (C.H.); zeng88liu@163.com (L.Z.); luwiz@oilcrops.cn (G.L.); fgp928076@hotmail.com (G.F.); chengyong58@yahoo.com.cn (Y.C.)

**Keywords:** rapeseed (*Brassica napus* L.), waterlogging, DGE (digital gene expression), roots, transcriptome

## Abstract

Although rapeseed (*Brassica napus* L.) is known to be affected by waterlogging, the genetic basis of waterlogging tolerance by rapeseed is largely unknown. In this study, the transcriptome under 0 h and 12 h of waterlogging was assayed in the roots of ZS9, a tolerant variety, using digital gene expression (DGE). A total of 4432 differentially expressed genes were identified, indicating that the response to waterlogging in rapeseed is complicated. The assignments of the annotated genes based on GO (Gene Ontology) revealed there were more genes induced under waterlogging in “oxidation reduction”, “secondary metabolism”, “transcription regulation”, and “translation regulation”; suggesting these four pathways are enhanced under waterlogging. Analysis of the 200 most highly expressed genes illustrated that 144 under normal conditions were down-regulated by waterlogging, while up to 191 under waterlogging were those induced in response to stress. The expression of genes involved under waterlogging is mediated by multiple levels of transcriptional, post-transcriptional, translational and post-translational regulation, including phosphorylation and protein degradation; in particular, protein degradation might be involved in the negative regulation in response to this stress. Our results provide new insight into the response to waterlogging and will help to identify important candidate genes.

## 1. Introduction

Waterlogging is one of the most widespread abiotic determinants for crop growth, leading to the depletion of oxygen, which is vital to plants [1]. The depletion of oxygen is a major feature of waterlogging because the diffusion of oxygen in water is 10^−4^ times slower than that in air [2]. The imbalance between the slow diffusion of gases and the rate that oxygen is consumed by micro-organisms and plant roots drastically reduces the supply of oxygen [3], which is vital to the roots of plant.

During recent years, gene expression studies in Arabidopsis [4–7], maize [8,9], rice [10,11], and other species [12–14] exposed to low oxygen have demonstrated that low oxygen stress causes drastic changes in gene expression. Although the expression of a majority of global genes was depressed, the accumulation of mRNAs was revealed for many genes under hypoxia. These genes included anaerobic proteins (ANPs) involved in sugar phosphate metabolism [15]. Studies have subsequently identified signal transduction components that are involved in the activation of *RopGAP4* (*Rop GTPase activating protein4*) [2,16], and transient induction of mitochondrial *alternative oxidase* (AOX), induction of calmodulin and CAP (calmodulin-associated peptide) [15,17,18]. Moreover, the induction of plant growth regulators under waterlogging stress are involved in signaling cascades that influence cellular responses, including increases in ethylene [19,20], abscisic acid (ABA) [21–25], gibberellic acid (GA) [26], and auxin (IAA) [27,28] and a reduction in cytokinin (CK) [29,30]. Transcriptional factors (TFs) play an extremely important role in waterlogging tolerance. In rice, two TFs, Snorkel [31] and Submergence-1A [32], have been cloned by mapped based cloning, and both of them encode ethylene-responsive factor-type transcription factors that have evolved opposite functions to adapt to different types of flood. In Arabidopsis, studies have revealed that oxygen sensing is mediated by group VII ERF (ethylene response factor) TFs through the N-End rule pathway [33].

Although many transcriptomic studies on waterlogging have addressed similar topics with regard to gene expression in response to waterlogging, this response has proven to have a very complex mechanism. Indeed, understanding the mechanisms that coordinate the regulation of waterlogging tolerance remains a fundamental challenge. Furthermore, there is still no report of a large-scale of gene expression analysis of the response to waterlogging in rapeseed (*Brassica napus* L.).

Rapeseed is particularly sensitive to waterlogging. The plants experience waterlogging when directly sown in paddy field planted as a rotation crop following rice in China, the largest rapeseed-planting country in the world [29,30]. Because there is a need to understand the response to waterlogging in rapeseed, it is necessary and helpful to study expression profiles under waterlogging in a tolerant variety of rapeseed.

To gain comprehensive insight into how rapeseed responds to waterlogging and to identify the genes important in mounting a response of waterlogging tolerance, here we report a detailed analysis of gene expression profiling in ZS9, a waterlogging-tolerant variety [29,30], at the vegetative growth stage under waterlogging using digital gene expression (DGE) method, a powerful tool for studying high-throughput gene expression profiling [34,35]. We identified sets of positively and negatively significantly expressed genes in response to waterlogging. Our analysis suggests that waterlogging affects a broad spectrum of functional categories and that the regulation of waterlogging tolerance is complex, involving with multiple levels of regulation. The mechanism of the response to waterlogging is discussed.

## 2. Results and Discussion

### 2.1. Results

#### 2.1.1. Analysis of DGE Libraries

To identify genes in response to waterlogging, RNA libraries were generated using the roots of ZS9 seedlings at 0 h (the control) and 12 h after waterlogging (the treatment) (Table 1). More than 30 million original sequencing tags were produced, representing 13,457,553 and 19,621,584 raw reads from the library of 0 h and 12 h, respectively. The junk tags were filtered (low quality tags, tags with one copy, tags containing N and tags of low quality) prior to mapping these tag sequences to the reference sequences, producing approximately 13.3 and 19.4 million clean sequence tags, respectively. For the two libraries, 57.32% and 60.31% of the clean tags were mapped unambiguously, with 4,221,453 (31.78% of the clean tags) and 6,684,936 (34.52% of the clean tags) clean tags being perfectly mapped with a stringent criterion of 0 mismatches within the 16-nucleotide tag alignments. Some 3.94% and 4.71% of the clean tags mapped to duplicated genes, alternate transcripts, or repeated sequences. Lastly, 30,964 and 28,954 unique genes representing 7,614,486 and 11,679,339 DGE tags from the control and the treatment libraries were obtained, and the counts for each unique gene were normalized to the reads per kb per million reads (RPKM) for the two libraries.

#### 2.1.2. Changes in Global Gene Transcription under Waterlogging

To characterize the genes involved in the response to waterlogging in roots at the seedling stage, the expression profiles at 12 h (the treatment) and 0 h (the control) of waterlogging were compared. A statistical analysis of the frequency of genes identified 4432 differentially expressed genes under waterlogging (Supplementary Data 1).

**Figure 1:**
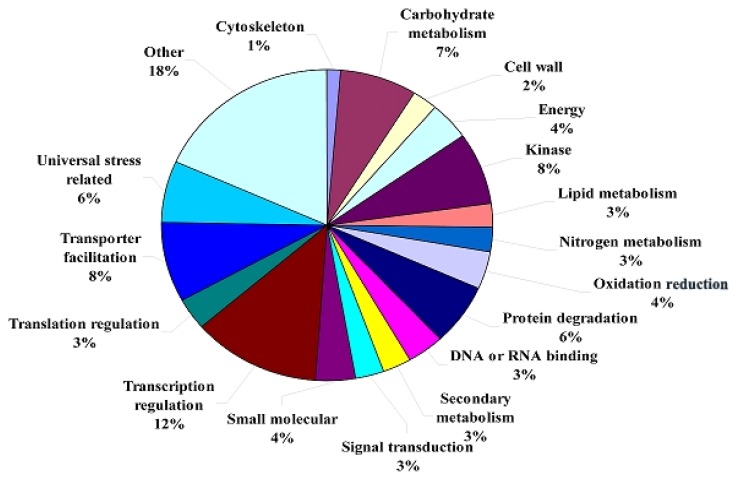
Functional categorization of all the annotated differentially expressed genes. This analysis was based on 2387 annotated genes, not including genes with “unknown function” or “no homology”.

An annotation analysis revealed that nearly half (45.6%, 2019/4432) of the differentially expressed genes were “functional unknown”, annotated as “unnamed protein product”, “hypothetical protein” or “unknown protein”. Moreover, 26 genes, accounting for 0.6% of all the differentially expressed genes, did not match to known sequences, defined as “no homology”, which suggested our study may allow for the identification of novel genes in the response to waterlogging tolerance. Based on GO, 2387 annotated genes were categorized into 18 functional categories (Figure 1). The largest categories were “transcription regulation” (12.4%), “transporter facilitation” (8.3%), and “kinase” (7.6%). The expected group associated with “carbohydrate metabolism” represented for 178 genes. In addition to “carbohydrate metabolism”, genes related to “lipid metabolism” (61 genes) and “nitrogen metabolism” (60 genes) were also identified, representing for 2.6% and 2.5%, respectively. As we expected, 151 genes (6.5%) were found to be categorized into the group of “universal stress related”. We also noticed a high percentage of genes related to “protein degradation” (152 genes). Interestingly, 83 genes (3.5%) involved in “DNA or RNA binding” were found to be differentially expressed under waterlogging. Additionally, the categories related to “energy” and “small molecular” each contained 92 genes (3.9%. A total of 93 genes (3.9%) involved in “oxidation reduction” were found. In addition to the regulation of transcription in response to waterlogging revealed by the largest group of genes related to “transcription regulation”, a set of 82 (3.4%) differentially expressed genes were involved in “translation regulation”. Substantial sets also included the groups of “signal transduction” (3.0%) and “secondary metabolism” (2.7%). 35 genes involved in “cytoskeleton” and 58 genes related to “cell wall” were also identified. A lot of significant genes (18.4%) from a wide variety of pathways were affected by waterlogging and were categorized into the group of “other function”.

#### 2.1.3. Transcriptomic Comparison of the Roots under Waterlogging Treatment and the Control Using DGE Tag Profiling

**Figure 2:**
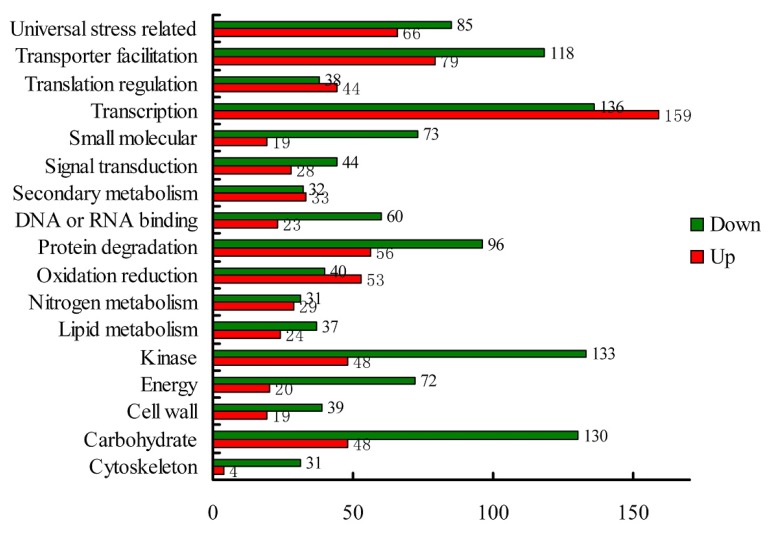
Comparison between up-regulated and down-regulated genes based on function categories.

**Figure 3:**
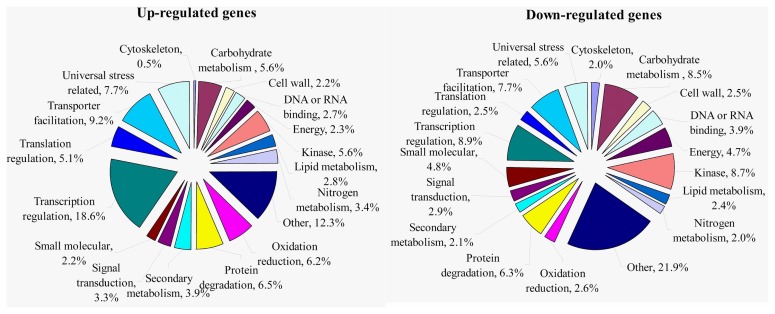
Functional categorization of up-regulated and down-regulated expressed genes. This analysis did not include the genes with “no function annotation” or “no homology”.

Of the 4432 differentially expressed genes, 1709 genes were up-regulated and 2723 genes were down-regulated. Based on the categorization of up-regulated and down-regulated genes respectively, we performed a comparison between these categories. Since there were more genes down-regulated under waterlogging compared that up-regulated as expected, there were more down-regulated genes than up-regulated ones in most categories (Figure 2). However, there were more genes up-regulated in response to waterlogging in the four categories of “oxidation reduction”, “secondary metabolism”, “transcription regulation”, and “translation regulation”. Additionally, only six genes with no homology were identified as being down-regulated under waterlogging, whereas there were up to 20 novel genes up-regulated in response to this stress. To provide further valuable information, gene Ontology (GO) assignments were performed based on the annotated genes up-regulated and down-regulated genes, respectively. As shown in Figure 3, the top three largest groups of up-regulated genes were “transcription regulation”, “transporter facilitation”, and “universal stress related” under normal conditions, while under waterlogging, “transcription regulation”, “kinase”, and “carbohydrate metabolism” were the top three largest groups. Surprisingly, genes related to “transcription regulation” comprised up to 18.6% of the up-regulated genes.

#### 2.1.4. Expression Levels of Differentially Expressed Genes under the Control and Waterlogging Stress

High-throughput sequencing can provide information about gene expression levels, and we observed that the majority of transcripts were represented by a few genes with abundant counts. Considering the abundance of all the differentially expressed genes under the control and the stress, the transcriptome at 0 h (the control) and 12 h (the treatment) under waterlogging condition consist of unevenly distributed sequence abundance in which the top 200 unique genes with the highest expression level accounted for 14.7% and 32.6% of the total counts of transcripts, respectively (Supplementary Data 2). The 200 most highly expressed genes in both samples were grouped based on their function (Table 2). 144 out of the top 200 abundant genes under the normal condition were down-regulated when waterlogging occurred, while under waterlogging up to 191 ones were those genes induced in response to the stress. Although most of the highly expressed genes under the control were down-regulated in response to the stress, the pathways of “universal stress related”, “carbohydrate metabolism”, “translation regulation”, and “energy” were enriched under waterlogging.

#### 2.1.5. Verification of The DGE Data by Real-Time Quantitative RT-PCR

To validate the results of the DGE data, the transcriptional level of 12 unigenes were examined by real time PCR. Although the change fold did not exactly match the number revealed by the DGE data for these genes, all the unigenes showed consistent expression patterns that were consisted with the DGE data, and exhibited > 2 fold higher expression in response to waterlogging (Table 3). The ANP encoding genes, including *glyceraldehyde-3-phosphate dehydrogenase* and *alcohol dehydrogenase*, were chosen in the real time PCR. The results showed that these two genes were up-regulated significantly under waterlogging indicated by both DGE data and verified by real time PCR, indicating that our experimental results for conditions of waterlogging were valid.

### 2.2. Discussion

The technology of DGE by high-throughput sequencing is now in common in use for transcriptomic analyses. In this study, we applied this method to evaluate gene expression under waterlogging in the roots of seedlings for rapeseed. As expected, many genes were differentially expressed when waterlogging occurred; some of these genes were further confirmed by real-time PCR experiments, demonstrating the validity of the DGE data. The GO analysis revealed that these differentially expressed genes were distributed among various pathways, suggesting that a wide spectrum of physiological processes was affected by the low-oxygen stress.

Although a relatively large number of genes were down-regulated by waterlogging, the pathways associated with “secondary metabolism”, “oxidation reduction”, “transcription regulation”, and “translation regulation” were enhanced under waterlogging, which confirms the current knowledge of the waterlogging response. It is apparent that a battery of signaling molecules, such as ethylene, ABA, GA, IAA and reduction of CK, is modulated during stressful conditions, and it is not surprising that the pathway of “secondary metabolism” was enhanced under waterlogging [21–30]. Moreover, ROS (reactive oxygen species) production has been suggested to be a component of signaling under hypoxia [36–38]. Genes responsible for ROS handling, such as *cytochrome c oxidase*, *peroxidase*, and *NADH-ubiquinone oxidoreductase*, *etc.*, were identified in our study, which is consistent with the previous report that the induction of oxidative metabolism is necessary for the induction of adaptive responses to waterlogging [36,39–41]. The pathways of “transcription regulation” and “translation regulation” were enhanced under the imposed stress, suggesting regulation of gene expression at both the transcriptional and translational levels, as discussed below.

#### 2.2.1. Protein Degradation May Be Involved in Negative Regulation in the Response to Waterlogging

Under normal conditions, 0 h (the control) in our study, the genes for development (called “developmental genes” herein) in various pathways (Table 2) were among the 200 most abundant genes and were distributed almost all categories. However, these “developmental genes” were down-regulated upon waterlogging. In our study, 2387 genes were reduced in response to waterlogging. The ratio of the number of reduced genes compared to induced genes was more than 1.5. Additionally, the expression level decreased for 72% of the 200 most highly expressed “developmental genes”, with only 10 of these genes remaining in the list of the 200 most highly expressed genes under waterlogging. This result is accordance with similar studies in Arabidopsis, rice, maize and other species. It is a widely accepted fact that the genes encoding enzymes for development under normal conditions, such as *phosphoenolpyruvate carboxylase*, *pyrophosphate-fructose-6-phosphate 1-phosphotransferase* and *glucose-6-phosphate dehydrogenase* (related to aerobic respiration) [9,39], which are not necessary for survival under waterlogging stress, are down-regulated when waterlogging occurs.

When a plant grows under normal conditions, different proteins encoded by different genes are expressed at different development stages, and transcription of the genes related to the previous stage should be down-regulated when the plant enters the next development stage, and the existing proteins should be degraded because they are no longer required. As showed in our study, 4% of the 200 most highly expressed genes at 0 h (the control) (Table 2) were genes involved in protein degradation and should play an important role in degrading the proteins encoded by “developmental genes” under normal conditions. As mentioned by Zou [8], the degradation of aerobic proteins would help decrease the consumption of oxygen and supply free amino acids for breakdown of carbon skeletons for the supply of energy under waterlogging. In our study, 56 genes were up-regulated in response to waterlogging (Figure 2) and may be involved in the above pathways.

Recently, a breakthrough in the study on waterlogging tolerance revealed that the N-end rule pathway of protein degradation acts as a homeostatic sensor of severely low oxygen levels in Arabidopsis through the regulation of key hypoxia-response TFs [33,42,43]. To some extent, the mechanism in Arabidopsis is similar to that in animals, in which the proteasomal degradation of key transcription factor hypoxia-induced factors (HIFs) depending on the proline hydroxylation controls the global response to hypoxia [44]. In other words, protein degradation plays a negative role in response to hypoxia. In our study, 96 genes were identified as being down-regulated by the stress, accounting for up to 6.3% of the reduced genes, some of which might be involved in the negative regulation of important protein in the response to waterlogging. In most transcriptomic studies under waterlogging, the up-regulated genes have more often been highlighted than the down-regulated genes based on the fact that hypoxia represses the expression of many genes. Indeed, very little is known about the role of gene repression in waterlogging tolerance. Our analysis includes the potential function of genes involved in protein degradation that were down-regulated, providing a new insight for future studies.

#### 2.2.2. Multiple Levels of Regulation Were Involved in the Response to Waterlogging

Plants respond to waterlogging in various ways.

Firstly, transcriptional regulation clearly plays an important role in response to waterlogging. Expression of 4432 genes was altered significantly under waterlogging in this study. Therefore, it is not surprising that the category of “transcription regulation” showed the largest number of differentially expressed genes, up to 12.4%. TFs have attracted considerable interest in previous studies. Some key genes regulating waterlogging that have been cloned in plants are TF encoding genes, including *Sub1A*, *Snorkel1*, *Snorkel2*, *HRE1*, *HRE2*, and *RAP2.2* [33,42,43,45], and, in particular, ethylene response factor (ERF). In the present study, seven genes coding ERFs were identified as being regulated, and five were induced: including *ERF2*, *ERF4*, *ERF7*, *ERF11*, and *ERF54*. The importance of these genes will require further verification.

Secondly, some reports have demonstrated that miRNA and alternative splicing play important regulation roles in response to waterlogging [46–48]. To support this, among the induced genes identified under waterlogging in this study, there was a category containing a considerable number of genes related to “DNA/RNA binding” (Figure 1), such as *RNA recognition motif* (*RRM*)*-containing protein*, *RNA-binding protein-like protein*, and *RNA-binding protein cp31*, which might be involved in the post-transcriptional regulation that occurs with this stress.

Thirdly, a variability in the efficiency of protein synthesis for different genes under waterlogging have been reported before [49] and is based on differing abilities of the transcripts of different genes to associate with the translational complexes [39]. A total of 82 genes involved in “translation regulation” were differentially expressed in response to waterlogging in this study. With global decrease in protein synthesis, it is interesting to find that 44 genes related to translation were up-regulated, including *glutamyl-tRNA synthetase*, *translation initiation factor*, and *ribosomal protein L17 family protein*. Considering of the information above, a question remains whether there are different translational machineries serving for the transcripts of different genes, leading to different protein synthesis efficiencies. Under waterlogging, some translation machinery might regulate the synthesis efficiency of different proteins depending on the quantity of the translation complexes themselves, in addition to the regulation of transcript abundance.

Finally, the pathway of phosphorylation plays an important role in signal transduction through regulating phosphorylation of specific protein [8]. We found that the expression level of genes related to phosphorylation, including *purple acid phosphatase 17*, *tyrosine specific protein phosphatase family protein*, were altered under waterlogging, These genes might regulate the activity of target proteins though the regulation of their own abundance. Additionally, the protein degradation discussed above is also involved in the regulation of gene activity under waterlogging.

## 3. Experimental Section

### 3.1. Plant Materials and Waterlogging Treatment

ZS9 [29,30] with high waterlogging tolerance was used in this study. Seeds were germinated on moist filter paper. After three days, germinated seeds were individually transplanted to sand chambers. All the plants were grown with 16/8 h day/night cycles at 30 °C/22 °C and a light intensity of 500 μ·mol·m^−2^·s^−1^. Seedlings with two leaves were used. Uniform seedlings were selected and divided into two groups: one group was cultured with normal water supply as the control and the other was submerged in water with all leaves in air as the treatment (Figure S2). Roots treated for 12 h and roots of the controls were harvested at the same time, and were stored at −80 °C.

### 3.2. RNA Isolation

Total RNA was isolated using TRIzol (Invitrogen, California, CA, USA) according to the manufacture’s instructions followed by RNase-free DNase treatment (Takara, Dalian, China). RNA quantity and quality were assessed by a Nanodrop spectrophotometer and by agarose gel electrophoresis.

### 3.3. DGE-Tag Profiling

Two DGE libraries were constructed using total RNA of roots of seedlings waterlogged for 12 h and that of the control with Illumina’s Digital Gene Expression Tag Profiling Kit according to the manufacturer’s protocol (Version 2.1B). The two tag libraries underwent Illumina proprietary sequencing chip for cluster generation through *in situ* amplification and were deep-sequenced using Illumina Genome Analyzer. The image files generated were processed to produce digital-quality sequence data.

For the raw data, low quality tags, adaptor sequences, tags with unknown nucleotides N, empty reads, tags that were too short or too long, and tags with only one copy, were filtered to get clean reads. The types of clean tags were represented as the distinct clean tags. Subsequently, we classified the clean tags and distinct clean tags according to their copy number in the library and showed their percentage in the total clean and distinct tags, and analyzed saturation of the two libraries.

For annotation, all the tags were mapped to the reference sequences, including NCBI EST database of *Brassica napus* L., and unigenes of the Brassica oleracea Genomics Database because there is no genome sequence of *Brassica napus* L. and its genome (AC genome) is a polyploidy of *Brassica rapa* genome (A genome) and *Brassica oleracea* genome (C genome). Only no more than 1-bp nucleotide mismatch was allowed.

### 3.4. Identification of Differentially Expressed Genes

The expression level of each gene was normalized to RPKM based on the number of clean tags. Genes were deemed significantly differentially expressed with a *p*-value < 0.005, FDR < 0.01 and a relative change threshold of two-fold in the sequence counts across libraries. Functional classification of differentially expressed genes was carried out according to the functional categories of GO.

### 3.5. Quantitative Real-Time PCR Analysis

Three biological replications with two technique replications of total RNA were used for quantitative real-time PCR analysis. Total RNA was treated with RNase-free DNase. Reverse transcription of total RNA (5 μg) was performed with M-MLV RTase cDNA Synthesis Kit (Takara, Dalian, China).

Real time PCR was carried out using a CFX96 Real-Time System C1000 Thermal Cycler (Bio-RAD, Hercules, CA, USA) using SYBRGreen PCR Master Mix (Takara, Dalian, China). Primers were designed using PRIMER3 software [50] and were listed in Table S1. The expression of *actin* was used as a control. PCR amplification conditions and the data analysis were referred to Zou’s report [8].

## 4. Conclusions

In summary, based on several pieces of evidence derived from this study, we speculate that the response to waterlogging is mediated by the regulation of the levels of transcription, post-transcription, translation and post-translation, including phosphorylation and protein degradation. Certainly, it is better to reveal the changes in the real activity of genes under waterlogging through metabolite profiling [13,51–53], which can consider all the levels of regulation. However, because the study of genes transcription is more convenient and less consuming and expensive, this approach is still a good choice to study the abundance of transcripts from the beginning of regulation, which also affects other levels of regulation.

A major objective of our study was to reveal the mechanism of waterlogging tolerance. Our study has demonstrated that gene regulation in response to oxygen deprivation is mediated by transcription, post-transcription, translation, and post-translation, including phosphorylation and protein degradation; in particular, protein degradation might be involved in the negative regulation of the stress response. A large number of differentially expressed genes always leads to difficulty in the characterization of the genes that are actually related to waterlogging tolerance. Based on our analysis, the genes related to multiple levels of regulation might be good choice for further study. This analysis provides a good starting point for future functional studies.

## Figures and Tables

**Table 1:** Summary of the two DGE (digital gene expression) libraries.

	0 h		12 h	
**Raw Reads**	13,457,553		19,621,584	
**Clean Reads**	13,283,443		19,364,949	
**Mapped Reads**	8,138,298		12,591,493	
	61.27% (8,138,298/13,283,443)		65.02% (12,591,493/19,364,949)	
**Perfect match**	4,221,453		6,684,936	
	31.78% a	51.87% b	34.52% a	53.09% b
**≤2bp mismatch**	3,916,845		5,906,557	
	29.49% a	48.13% b	30.5% a	46.91% b
**Unique match**	7,614,486		11,679,339	
	57.32% a	93.56% b	60.31% a	92.76% b
**Multi-position match**	523,812		912,154	
	3.94% a	6.44% b	4.71% a	7.24% b
**Unmapped Reads**	5,145,145		6,773,456	
	38.73% a		34.98% a	

Additionally, the sequencing saturation was analyzed in the two libraries to estimate whether the sequencing depth was sufficient for the transcriptomic coverage. The genes mapped by all clean tags and unambiguous clean tags increased with the total number of tags. When the sequencing counts reached 2.5 million tags or higher, the number of detected genes was saturated (Figure S1), indicating that the sequencing depth was sufficient for both of the two libraries.

a was represented the ratio was from the comparison between the number of sequences and the number of clean reads;

b was represented the ratio was from the comparison between the number of sequences and the number of mapped reads.

**Table 2:** Distribution of the top 200 highly expressed genes under normal and waterlogging condition.

Categories	Control-total	% ^a^	Control-down	% ^b^	Control-up	% ^c^	Waterlogged-total	% ^d^	Waterlogged-up	% ^e^	Waterlogged-down
Unknown function	79	39.5%	60	41.7%	19	33.9%	84	42.0%	80	41.9%	4
Universal stress related	17	8.5%	10	6.9%	7	12.5%	20	10.0%	18	9.4%	2
Carbohydrate metabolism	13	6.5%	8	5.6%	5	8.9%	12	6.0%	12	6.3%	0
Transporter facilitation	10	5.0%	7	4.9%	3	5.4%	11	5.5%	10	5.2%	1
Other	15	7.5%	12	8.3%	3	5.4%	9	4.5%	9	4.7%	0
Translation regulation	8	4.0%	2	1.4%	6	10.7%	8	4.0%	8	4.2%	0
Protein degradation	8	4.0%	5	3.5%	3	5.4%	8	4.0%	8	4.2%	0
Oxidation reduction	6	3.0%	4	2.8%	2	3.6%	7	3.5%	7	3.7%	0
No homology	4	2.0%	1	0.7%	3	5.4%	7	3.5%	6	3.1%	1
Transcription regulation	4	2.0%	3	2.1%	1	1.8%	6	3.0%	6	3.1%	0
Nitrogen metabolism	5	2.5%	4	2.8%	1	1.8%	6	3.0%	6	3.1%	0
Lipid metabolism	3	1.5%	2	1.4%	1	1.8%	5	2.5%	5	2.6%	0
Kinase	3	1.5%	3	2.1%	0	0.0%	4	2.0%	4	2.1%	0
Signal transduction	5	2.5%	5	3.5%	0	0.0%	3	1.5%	2	1.0%	1
Secondary metabolism	5	2.5%	4	2.8%	1	1.8%	3	1.5%	3	1.6%	0
Energy	3	1.5%	2	1.4%	1	1.8%	2	1.0%	2	1.0%	0
DNA or RNA binding	0	0.0%	0	0.0%	0	0.0%	2	1.0%	2	1.0%	0
Cell wall	5	2.5%	5	3.5%	0	0.0%	2	1.0%	2	1.0%	0
Small molecular	2	1.0%	2	1.4%	0	0.0%	1	0.5%	1	0.5%	0
Cytoskeleton	5	2.5%	5	3.5%	0	0.0%	0	0.0%	0	0.0%	0
Total	200	-	144	-	56	-	200	-	191	-	9
Percentage^f^	-	-	72.0%		28.0%		-	-	95.5%		4.5%

^a^ and ^d^ represent for the percentage of genes accounting of the top 200 highly expressed genes under normal and waterlogging condition, respectively; ^b^ represents for the percentage of genes accounting of induced ones under normal condition, while ^c^ represents for reduced ones; ^e^ represents the percentage of genes accounting of induced ones under waterlogging; ^f^ represents the percentage of induced and reduced genes under normal and waterlogging condition.

**Table 3:** Verification of DGE results by real time PCR.

Gene ID	Annotation	Fold change by DGE ^a^	Fold change by Q-PCR
Bra038700	*polygalacturonase inhibitory protein*	3.2	9.8 ± 1.4
Bra021558	*nine-cis-epoxycarotenoid dioxygenase3*	3.1	4.9 ± 1.1
Bra003701	*AP2 domain containing protein RAP2.5*	2.5	7.4 ± 1.5
Bra014080	*hydrolase*	2.4	24.9 ± 1.1
Bra007609	*glycoside hydrolase family 28 protein*	2.3	12.2 ± 1.3
Bra016729	*glyceraldehyde-3-phosphate dehydrogenase 1*	4.1	6.4 ± 1.3
Bra022115	*transcription factor*	2.1	8.9 ± 1.2
Bra004778	*Stearoyl-acyl carrier protein desaturase*	17.0	73.0 ± 1.4
Bra012551	*abscisic acid 8′-hydroxylase/oxygen binding*	0.3	0.24 ± 0.01
Bra019528	*betaine aldehyde dehydrogenase*	0.3	0.3 ± 0.01
Bra015693	*alcohol dehydrogenase*	16.2	12.1 ± 1.2
Bra030945	*phosphoenolpyruvate carboxylase*	0.3	0.2 ± 0.03

The transcriptional level of candidate genes was examined by real time PCR with three biological replications of RNA and *actin* was used as an internal control.

## Supplementary Files

Supplementary File 1 Supplementary Information (PDF, 428 KB)

Supplementary File 2 Supplementary Table 1 (XLS, 1057 KB)

Supplementary File 3 Supplementary Table 2 (XLS, 107 KB)
