# Supplementary material for: The Transcriptome of Brassica napus L. Roots under Waterlogging at the Seedling Stage
Source: Int J Mol Sci. 2013 Jan 28;14(2):2637–51. doi: 10.3390/ijms14022637 (PMC3588007; doi:10.3390/ijms14022637)
Supplement: Supplementary File 1 — Supplementary Information (PDF, 428 KB) [file ijms-14-02637-s001.pdf]

Supplementary Information

Figure S1. The analysis of saturation of DGE for two libraries.

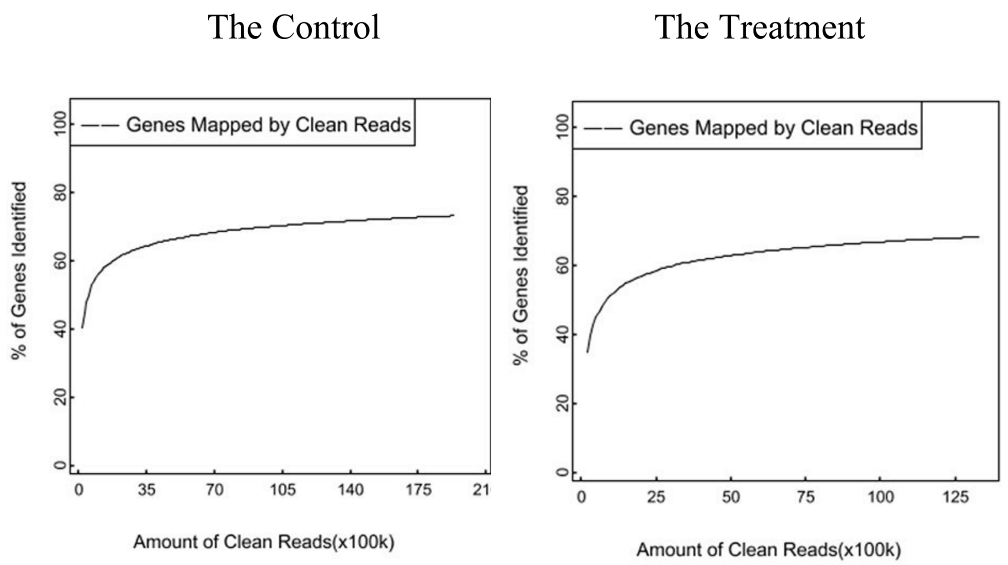

Figure S2. The procedure of sample preparation.

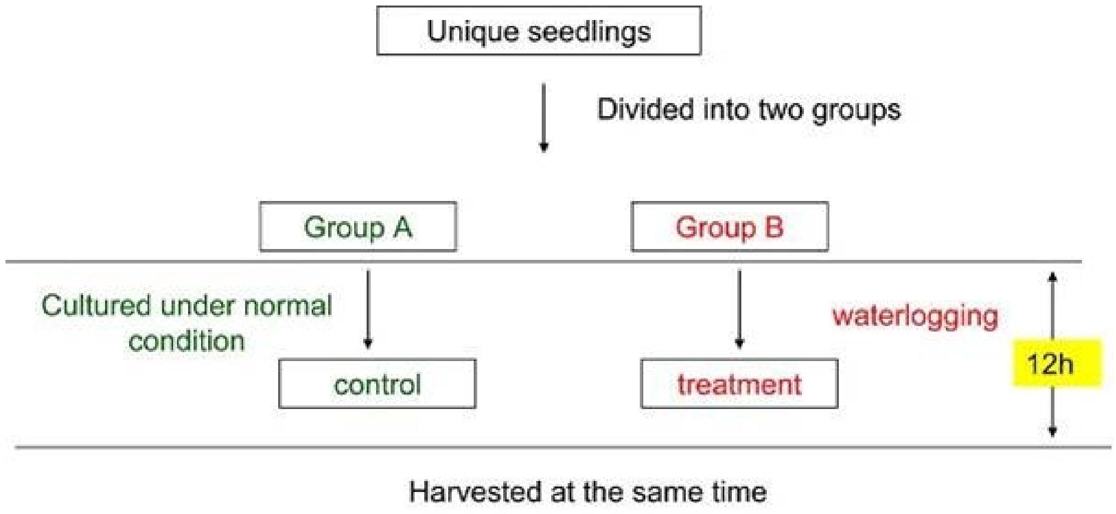

Table S1. Gene-specific primers for real-time PCR.

Gene ID	Annotation	Primes (5'->3')
Bra038700	*polygalacturonase inhibitory protein*	CGCGTCACCGCTCTCACCAT<br>CGGAGCTCAAGAAGGTCGCCG
Bra021558	*nine-cis-epoxycarotenoid dioxygenase3*	AGCCATCGGCGAGCTTCACG<br>TCCGTGCGCTGGGTCGACTA
Bra003701	*AP2 domain containing protein RAP2.5*	CGGTGGCGCGTCCTGTGTAT<br>CATCGGAGGCCGAGCAGCAC
Bra014080	*hydrolase*	TGGTGGCGATTCGTGTGCCC<br>TCGGCGACCCATCCGCAAAC
Bra007609	*glycoside hydrolase family 28 protein*	AGGCCTTCCAGAGCGCCGTA<br>TGAGCCATTTTCCGGCGGGG
Bra016729	*glyceraldehyde-3-phosphate dehydrogenase 1*	TGGGGTGAGGCTGGTGCTGA<br>GCAGCGGCCTTGTCCTTGTCA
Bra022115	*transcription factor*	GCGGCGACGGTGGAGAAACA<br>CCCCCACGGCCTCTGTCTCA
Bra004778	*Stearoyl-acyl carrier protein desaturase*	CCTGACGGCACTGTGGTGGC<br>GCTTGTCGTCACGCCCATCGT
Bra012551	*abscisic acid 8'-hydroxylase/ oxygen binding*	GCGGCGACGGTGGAGAAACA<br>CCCCCACGGCCTCTGTCTCA
Bra015693	*alcohol dehydrogenase*	CGAGAGGACACTCAGGGGTA<br>TGTACTTTTCGACCACCCCG
Bra019528	*betaine aldehyde dehydrogenase*	GCTGTAGGCTTGGTCCTGTT<br>GCACCTTCCTTCCTAGCGTT
Bra030945	*phosphoenolpyruvate carboxylase*	GAGTACTTCCGCCTCGCTAC<br>GCCGCTTGGTTTCCTCTTTG
	actin	ACGAGCTACCTGACGGACAAG<br>GAGCGACGGCTGGAAGAGTA

© 2013 by the authors; licensee MDPI, Basel, Switzerland. This article is an open access article distributed under the terms and conditions of the Creative Commons Attribution license (http://creativecommons.org/licenses/by/3.0/).
